# Insecticide resistance in phlebotomine sandflies in Southeast Asia with emphasis on the Indian subcontinent

**DOI:** 10.1186/s40249-016-0200-3

**Published:** 2016-11-07

**Authors:** Ramesh C. Dhiman, Rajpal S. Yadav

**Affiliations:** 1 National Institute of Malaria Research (ICMR), Delhi, 110077 India; 2 Department of Control of Neglected Tropical Diseases, World Health Organization, Geneva, Switzerland

**Keywords:** DDT, Alpha-cypermethrin, Indoor residual spraying, Indian subcontinent, Insecticide resistance, *Phlebotomus argentipes*, *Phlebotomus papatasi*, Sandflies, Visceral leishmaniasis

## Abstract

**Background:**

Visceral leishmaniasis, commonly known as kala-azar in India, is a global public health problem. In Southeast Asia, Bangladesh, Bhutan, India, Nepal, Sri Lanka and Thailand are endemic for visceral leishmaniasis. The role of sandflies as the vector of kala-azar was first confirmed in 1942 in India. Insecticide resistance in *Phlebotomus argentipes* Annandale and Brunetti, the vector of kala-azar in the Indian subcontinent, was first reported in 1987 in Bihar, India. This article provides a scoping review of the studies undertaken from 1959 to 2015 on insecticide resistance in *P. argentipes* and *P. papatasi* (Scopoli), the vectors of visceral and cutaneous leishmaniasis respectively, in Southeast Asia, mainly in Bangladesh, India, Nepal and Sri Lanka.

**Results:**

Studies undertaken in areas of Bihar and West Bengal in India where kala-azar is endemic have reported resistance of *P. argentipes* to DDT, while in non-endemic areas it has been reported to be susceptible. In areas of Nepal bordering India, there are indications of resistance to DDT; biochemical resistance has been reported in Sri Lanka. No laboratory studies have been undertaken in Bangladesh; however, the sandfly vector is reported to be still susceptible to pyrethroids in all kala-azar endemic areas in the aforementioned countries.

**Conclusions:**

Studies are needed to determine the resistance of sandfly vectors to all available classes of potential insecticides in kala-azar endemic areas. There is a need to assess the impact of indoor residual spraying with DDT and pyrethroids on the incidence of kala-azar in India where 54 districts remain endemic for the disease, strengthen entomological surveillance capacity, and develop and implement an insecticide management plan. Alpha-cypermethrin indoor residual spraying has been introduced in 33 kala-azar endemic districts in Bihar State of India in a pilot trial; the outcomes should be used to inform decisions on expanding coverage with alpha-cypermethrin in all remaining endemic districts to achieve the revised goal of elimination of visceral leishmaniasis by 2020.

**Electronic supplementary material:**

The online version of this article (doi:10.1186/s40249-016-0200-3) contains supplementary material, which is available to authorized users.

## Multilingual abstracts

Please see Additional file 1 for translations of the abstract into the five official working languages of the United Nations.

## Introduction

Phlebotomine sandflies are the vectors of leishmaniasis. Of the various manifestations of leishmaniasis in the world, two forms (visceral leishmaniasis (VL) and cutaneous leishmaniasis (CL)) are known from Southeast Asia. The role of sandflies as vectors of VL, commonly known as kala-azar in India, was established in 1942 by Swaminath et al. [1]. In Southeast Asia, the distribution of VL is confined to Bangladesh, Bhutan, India, Nepal, Sri Lanka and Thailand, while CL is confined to India and Sri Lanka [2]. In the Indian subcontinent the vector of kala-azar is *Phlebotomus argentipes* Annandale and Brunetti, while *P. papatasi* (Scopoli), *P. sergenti* and *P. salehi* are the vectors of CL. Sandflies are fragile tiny insects with poor wing venation; therefore, they prefer to hop and rest in the dark corners of houses and do not fly long distances. *P. argentipes* usually rests indoors in cattle sheds, human dwellings, and mixed dwellings of both human and cattle, while outdoor resting in tree holes and under culverts has also been reported. *P. papatasi* is usually found in association with *P. argentipes* while *P. salehi* is found in rodent burrows.

The indoor resting behaviour of sandflies makes them a suitable target for control by indoor residual spraying (IRS) with insecticides. As a result, control of malaria with DDT starting in 1950s immensely benefitted VL control in the Indian subcontinent. Until1978, sandflies were known to be susceptible to insecticides but resistance to dichlorodiphenyltrichloroethane (DDT) in *P. papatasi* and *P. argentipes* was reported in 1979 and 1990 [3, 4]. The spatial distribution of leishmaniaisis and vectors is increasing in response to changing ecological and climate change scenarios [5–12]. Furthermore, Bangladesh, India and Nepal have launched elimination programmes for VL [13]; therefore, it is imperative to know the latest status of susceptibility of vector species to insecticides being used by the national programmes in different regions.

This article reviews the current status of insecticide resistance in sandflies with emphasis on *P. argentipes*, the vector of VL in India, Bangladesh and Nepal, in order to identify research areas and adopt appropriate insecticides for vector control for effective implementation of VL elimination programmes.

## Review

### Material and methods

The literature search made through PubMed using ‘vector control’ and ‘sandflies’ as key words resulted in 714 publications. Thereafter, insecticide resistance and sandflies were used as key words, which resulted in a shortlist of only 54 publications. Using ‘control of *Phlebotomies argentines’*as the key word, 84 references were found while with the key words ‘leishmaniasis, insecticide resistance, and sandflies’, 169 references were found. None of the searches yielded all published papers on insecticide resistance in phlebotomine sandflies. The papers published from Southeast Asia in non-indexed journals were searched through published reviews and cross-references on insecticide resistance in phlebotomine sandflies. The papers not dealing with insecticide resistance, vector control, leishmaniasis and sandflies were excluded. After identifying the suitable title of the papers, the abstracts and full papers were extracted through the Google search engine, the libraries of the National Centre for Disease Control and the National Institute of Malaria Research. Only those papers dealing with the susceptibility or resistance status of sandflies to insecticides and impact on vector control of leishmaniasis were considered for review.

## Results

### Insecticide policy for vector control of visceral leishmaniasis

The main strategy for vector control of VL is to conduct two rounds of indoor residual spraying with DDT (1 g/m^2^) in human dwellings and cattle sheds up to a height of 6 ft. The first round is usually undertaken from February to March and the second round during May to June but may vary from state to state. In Bangladesh and Bhutan, pyrethroid insecticides are used; in Nepal, DDT and pyrethroids are used, while in India 50 % DDT (wettable powder) is used but in 2015, alpha-cypermethrin 5 % WP (synthetic pyrethroid) at 25 mg/m^2^ was introduced in seven pilot districts. In 2016, the plan is to cover 33 endemic districts in Bihar state. In accordance with the roadmap for elimination of kala-azar (www.nvbdcp.gov.in), micro-planning for vector control was instituted in 2014 whereby any village or hamlet reporting KA cases in the past 3 years qualifies for 100 % coverage by spraying.

### Studies of insecticide resistance in India

Kala-azar has been endemic in the Indian continent since 1824 and has caused devastating epidemics. During the initial years of the anti-malaria campaign in India (1953–1958) the incidence of kala-azar also declined sharply apparently due to the collateral benefit of IRS with DDT [14]. In 1979, resistance in *P. papatasi* [3] was confirmed from Muzaffarpur district of Bihar. No mortality of *P. papatasi* was recorded when exposing the sandflies to 4 % DDT for one hour (LC50 > 4 % × 24 h) whereas *P. argentipes* was susceptible (LC50 0.48 % × 1 h). The LC50 value for dieldrin was 0.32 % × 1 h for *P. papatasi* and 0.16 % for *P. argentipes*. Thereafter, interest in studying the susceptibility of sandflies to insecticides, particularly to DDT, grew in areas endemic for kala-azar and reports began to arrive after 1979.

**Fig. 1:**
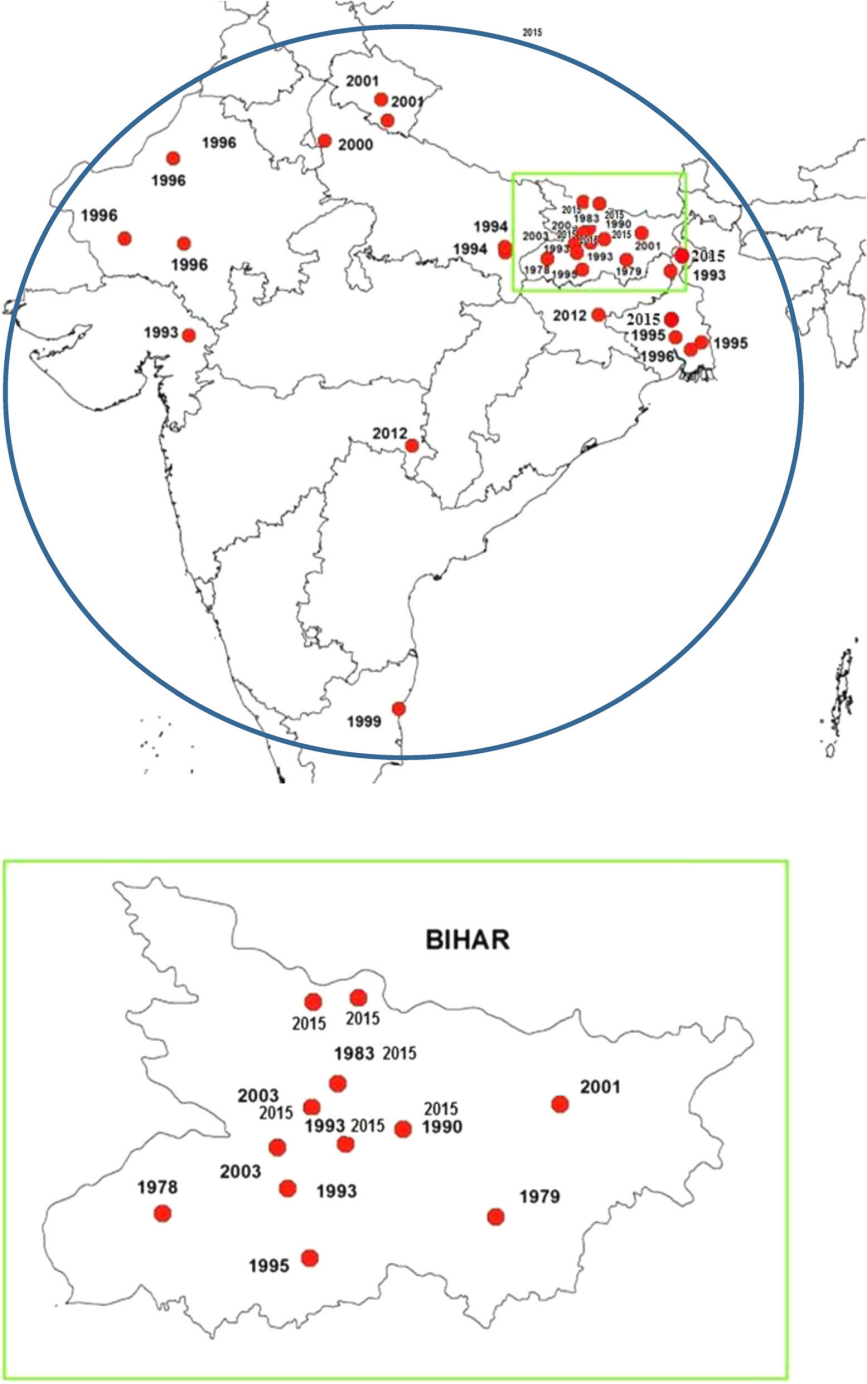
Locations in India (top) and Bihar state ( bottom) where susceptibility tests against  sandflies have been reported  since 1978

The geographical locations of studies undertaken on susceptibility of sandflies to DDT or other insecticides are given in Fig. 1. Susceptibility of *P. argentipes* to DDT was also studied in West Bengal in 1959 [15] when the sandflies were found to be fully susceptible (95–100 %). Kaul et al. [16] published preliminary findings on the susceptibility status of *P. argentipes* and *P. papatasi* collected from Bihar; *P. argentipes* was found to be susceptible and *P. papatasi* to be resistant with LC50 values from 0.5 to 0.6 × 1 h for *P.argentipes* but > 2 % × 1 h for *P. papatasi*. In 1979 detailed results were published by Joshi et al. [3] who confirmed the presence of resistance in *P. papatasi.* These two studies led to a realization of the problem of resistance in sandflies in India; thereafter many studies were undertaken in different parts of India following standard procedure [17], the findings of which are summarized in Table 1.

**Table 1:** Status of insecticide resistance in phlebotomine sandflies in the Southeast Asia region

Geographical area	Sandfly species	Insecticide	Result	Reference
A. Bangladesh	*P. argentipes*	DDT	Susceptible	Choudhury (2000) [41]
B. India				
Muzaffarpur District (Bihar)	*P. argentipes*	DDT	Susceptible	Kaul et al. (1978) [16]
		dieldrin		
Muzaffarpur and Samastipur districts (Bihar)	*P. papatasi*	DDT	Resistant	
		dieldrin	Susceptible (in both districts)	
Muzaffarpur District (Bihar)	*P. papatasi*	DDT	Resistant	Joshi et al. (1979) [3]
West Bengal	*P. argentipes*	DDT	Susceptible	Sen (1959) [15]
Muzaffarpur District (Bihar)	*P. papatasi*	DDT	Resistant	Dhanda et al. (1983) [18]
Samastipur District (Bihar)	*P. argentipes*	DDT	Tolerant	Mukhopadhyay et al. (1990) [4]
Begusarai and Muzaffarpur districts (Bihar)	*P. papatasi*	DDT	Resistant in both districts	Das et al. (1995) [19]
Ghaziabad District (Uttar Pradesh) and Midnapur District (West Bengal)	*P. papatasi*	DDT	Resistant	Dhiman and Mittal (2000) [20]
		dieldrin	Resistant	
		malathion	Resistant	
		deltamethrin	Suceptible (Populations of both districts)	
Panchmahal District (Gujarat)	*P. papatasi*	DDT and dieldrin	Susceptible	Thapar et al. (1993) [21]
Bikaner District (Rajasthan)	*P. papatasi*	DDT, dieldrin and propoxur	Resistant	Bansal and Singh (1996) [22]
		malathion, fenitrothion and permethrin	Susceptible	
Pali and Barmer districts (Rajasthan)	*P. papatasi*	DDT	Resistant	Singh and Bansal (1996) [23]
		dieldrin, malathion, fenitrothion and propoxur	Susceptible	
Vaishali District	*P. argentipes*	DDT	Resistant (15.4 % mortality)	Kaul et al. (1993) [25]
Patna District (Bihar)	*P. papatasi*		Resistant (2.9 % mortality)	
	*P. argentipes*		Susceptible (100 % mortality)	
Varanasi District (Uttar Pradesh)	*P. argentipes*	DDT	Susceptible	Joshi and Rai (1994) [28]
	*P. papatasi*		Susceptible	
West Bengal	*P. argentipes*	DDT	Susceptible	Mukhopadhyay et al. (1996) [29]
	*P. papatasi*		Resistant	
Sahibganj District (Bihar)	*P. argentipes*	DDT	Resistant	NMEP (1991) [30]
		dieldrin	Susceptible	
24 Parganas District (West Bengal)	*P. argentipes*	DDT	Resistant	Basak and Tandon (1995) [31]
Hoogly District (West Bengal)	*P. argentipes*	DDT	Susceptible	Chandra et al. (1995) [32]
Maldah District (West Bengal)	*P. argentipes*	DDT	Resistant (40–61.5%mortality)	Kumar et al. (2015) [33]
Pondicherry	*P. argentipes*	DDT	Tolerant	Amalraj et al. (1999) [34]
		BHC	Tolerant	
		malathion	Resistant	
		deltamethrin	Resistant	
		permethrin	Resistant	
		bendiocarb	Susceptible	
	*P. papatasi*	DDT	Tolerant	
		BHC	Susceptible	
		malathion	Tolerant	
		deltamethrin	Tolerant	
		permethrin	Resistant	
		bendiocarb	Susceptible	
Vaishali	*P. argentipes*	DDT	Resistant (71–78 % mortality) to almost susceptible (97.57 % mortality)	Singh et al. (2001) [35]
Darbhanga			Susceptible (98.24 % mortality) to tolerant (96.28 % mortality)	
Patna and Samastipur districts (Bihar)			Susceptible (100 % mortality)	
Nainital and Almora districts (Uttarakhand)	*P. argentipes*	DDT	Susceptible	Rao et al. (2001) [40]
Vaishali District	*P. argentipes*	DDT	Resistant	Dhiman et al. (2003) [36]
		deltamethrin	Susceptible	
Patna District (Bihar)		DDT	Susceptible	
		malathion	Susceptible	
Muzaffarpur, Vaishali and Patna districts combined (Bihar)	*P. argentipes*	DDT	Resistant (43 % mortality)	Dinesh et al. (2010) [37]
		deltamethrin	Susceptible (95–100 % mortality)	
Gadchiroli District (Maharashtra), Ramgarh District (Jharkhand), Katihar and Vaishali districts (Bihar)	*P. argentipes*	DDT	Resistant	Singh et al. (2012) [39]
		malathion	Susceptible	
		deltamethrin	Susceptible	
Patna District (Bihar)		DDT	Verification required (89 % mortality)	
		malathion and deltamethrin	Susceptible	
Patna	*P. argentipes*	DDT	Resistant, tolerant and susceptible	Singh and Kumar (2015) [38]
Vaishali			Resistant, tolerant	
Muzaffarpur				
			Resistant, tolerant and susceptible	
Samastipur, Sheohar and Sitamarhi districts (Bihar)			Resistant	
			Susceptible	
			Resistant	
C. Nepal				
Dhansua District	*P. argentipes*	DDT	Susceptible to both insecticides	Anonymous (2000) [43]
	*P. papatasi*	malathion		
Dhansua District	*P.argentipes*	malathion, bendiocarb, deltamethrin and lambda-cyhalothrin	Susceptible to all insecticides	Environmental Health Project (2001) [42]
Sunsari and Morang districts	*P. argentipes*	DDT	Resistant (62%mortality) in bordering area with India, otherwise susceptible in other areas	Dinesh et al. (2010) [37]
		deltamethrin	Susceptible (96–99 % mortality)	
D. Sri Lanka				
Delft islands	*P. argentipes*	malathion	Biochemical evidence of resistance	Surendran et al. (2005) [44]

Dhanda et al. [18] tested susceptibility of *P. papatasi* in Muzaffarpur district, Bihar, India and found only 14.15 % mortality with 4 % DDT while 82.7–95 % with 5 % malathion papers with 1 hour exposure. As most of the control measures were directed towards *P. argentipes*, the vector of kala-azar, the findings did not influence any change to the strategy for kala-azar control. Dasgupta et al. [19] also corroborated high degree of resistance in *P. papatasi* (no mortality out of 170 sandflies tested) from Begusarai and Muzaffarpur districts in Bihar.

Using WHO test papers Dhiman and Mittal [20] evaluated resistance in F1 generation of *P. papatasi* collected from Midnapur districts in West Bengal and Ghaziabad district, Uttar Pradesh, India. *P. papatasi* showed a high degree of resistance to both 4 % DDT (16.7 % mortality in Ghaziabad and 75 % mortality in West Bengal) and 5 % malathion (58.6 % in West Bengal).

In Panchmahal district of Gujarat, India only 9.2 % mortality against 0.4 % dieldrin (with 2 h exposure) and 18 % mortality with 4 % DDT in *P. papatasi* was reported [21]. In Bikaner, Rajasthan (India) a high degree of resistance in *P. papatasi* to DDT, dieldrin and propoxur was reported while full susceptibility to malathion, fenitrothion and permethrin [22].

In a study in Pali and Barmer districts of Rajasthan, India, Singh et al. [23] reported that *P. papatasi* was resistant to 4 % DDT (79.5 % mortality) but susceptible to dieldrin, malathion, fenitrothion and propoxur. Since DDT had been extensively used in the area, the result indicated development of resistance due to insecticide pressure.

Mukhopadhyay et al. [24] observed resurgence of *P. argentipes* and *P. papatasi* sandflies in northern Bihar following indoor residual spraying with DDT and provided a clue to the possible development of resistance in sandflies. Later on, Mukhopadhyay et al. [4] for the first time reported development of tolerance in *P.argentipes* from Samastipur district in Bihar.

After the report of tolerance in *P. argentipes* to DDT, studies were undertaken on the impact of DDT house spraying on field populations of the vector species in Bihar, Uttar Pradesh and West Bengal in India and in Bangladesh and Nepal [25–27].

Joshi and Rai [28] studied the impact of DDT spraying on field populations of *P. argentipes* and *P. papatasi* in Varanasi district, India (1987–1988) and found that *P. argentipes* was susceptible to DDT and “in the absence of selection pressure even *P. papatasi* appears to be sensitive to DDT”.

In West Bengal, *P. argentipes* was found susceptible to DDT while *P. papatasi* was resistant in the field as well as under laboratory conditions [29]. Using 4 % DDT papers, 0–96 % mortality in *P. papatasi* was recorded in different areas*.*

In 1991, the National Malaria Eradication Programme of India reported 82–100 % mortality in *P. argentipes* collected from Sahibganj district (Jharkhand, formerly a part of Bihar state) against DDT test papers [30].

Basak and Tandon [31] and Chandra et al. [32] found resistance in *P.argentipes* from 24 Parganas (West Bengal), India while 100 % susceptibility from Hoogly district of West Bengal. Recently, in a study undertaken in two villages of Maldah district, West Bengal, India, Kumar et al. [33] found 40–61.5 % mortality of *P. argentipes* against 4 % DDT indicating development of resistance.

Amalraj et al. [34] reported tolerance in *P. argentipes* and *P. papatasi* from Pondicherry, southern India against DDT and malathion but resistance to permethrin. The study also suggested that bendiocarb, a carbamate compound, may be used against populations of *P. argentipes* resistant to organophosphates and pyrethroids.

In 2001, various levels of susceptibility of *P. argentipes* to DDT were reported by Singh et al. [35]: 71–78 % mortality in Vaishali district to 100 % mortality in Patna and Samastipur districts and borderline resistance in sandfly populations of Darbhanga and other parts of Vaishali district, where verification of the resistance levels was suggested.

Dhiman et al. [36] also reported resistance in *P. argentipes* from Vaishali district, Bihar to DDT while susceptibility to malathion and deltamethrin. Dinesh et al. [37] reported 43 % mortality with 4 % DDT in *P. argentipes* collected from three districts of Bihar. A recent study undertaken in 42 villages of six districts of Bihar in India [38] revealed that *P. argentipes* has developed resistance to DDT, susceptible to tolerance to malathion and full susceptibility to deltamethrin. Interestingly, this study showed even 100 % susceptibility of *P. argentipes* to DDT in a few villages in Patna and Muzaffarpur districts that are less endemic for VL and thus not exposed to insecticide pressure.

In other parts of India, Singh et al. [39] reported resistance to DDT (89.5 % mortality) in *P. argentipes* from Gadchiroli (Maharashtra), Ramgarh (Jharkhand), and Lalganj and Patna (Bihar, India) and full susceptibility to malathion and deltamethrin.

Rao et al [40] found *P. argentipes* to be highly susceptible (98–100 % mortality) to DDT in Nainital and Almora districts of Uttarakhand.

### Studies in Bangladesh, Bhutan, Nepal and Sri Lanka

In Bangladesh, *P. argentipes* has been reported to be susceptible to DDT [41]. In Nepal, the same vector species is susceptible to DDT based on studies undertaken in Dhansua district [42, 43] where *P. papatasi* was also found to be susceptible to DDT and malathion. A review by the Environmental Health Project [42] also reported full susceptibility of *P.argentipes* to malathion, bendiocarb, deltamethrin and lambda-cyhalothrin. Dinesh et al. [37] found resistance in *P. argentipes* (only 62 % mortality in villages of Sunsari district, Nepal); otherwise sandflies were fully susceptible to DDT. However, with 0.05 % deltamethrin, 96–100 % mortality was found in both India and Nepal.

In Sri Lanka, Surendran et al. [44] provided biochemical evidence (through elevated levels of esterases) of development of resistance in *P. argentipes* to malathion, the insecticide that was being used for malaria vector control.

### Experiences with operational control of sandflies

The effectiveness of indoor residual spraying on kala-azar for control of *P. argentipes*/VL has been reported from India and to some extent from Nepal. Regarding the usefulness of long-lasting insecticide nets in control of sandfly populations, one study from India found no reduction in density of female sandflies [45]. However, a cluster randomized trial showed that village-wide use of long-lasting insecticide nets reduced the density of sandfly vectors up to 25 % and recommended the use of treated nets as part of VL control programmes [46]. In Nepal, Das et al. [47] found that indoor residual spraying and use of long-lasting insecticide nets were both effective in significantly reducing the density of sandfly vectors.

In Bihar, resurgence of *P. papatasi* was reported one month after DDT spraying while *P. argentipes* reappeared after 6 months of spraying [24]. Kaul et al [25] monitored the impact of DDT spraying on field populations of *P. argentipes* and *P. papatasi* in Vaishali and Patna districts. Although the impact of DDT was found to significantly reduce the density of sandflies between sprayed and unsprayed villages, the susceptibility tests using 4 % DDT showed only 15.4 % mortality of *P. argentipes* and 2.9 % mortality of *P. papatasi*. The study established field evidence for the development of resistance in *P. argentipes* to DDT in Bihar basically due to selection pressure of IRS rounds. In West Bengal, *P. argentipes* reappeared 9 months after spraying and *P. papatasi* within one month of spraying [29]. Kumar et al. [48] while monitoring the density of *P. argentipes* in two districts each in North and South Bihar representative of high and low endemicity for VL, observed that in North Bihar, man hour density (that is, the number of sandflies collected by one person in one hour) of vector species ranged from 5.36 to 10.96 and in South Bihar from 11.20 to 21.40. The reason for this difference was attributed to frequent DDT spraying in North Bihar. Picado et al. [46] found that use of LN in India and Nepal reduced the density of sandfly vectors by up to 25 % and recommended that LN could be used as part of the VL control programme. In Nepal, Das et al. [47] found the usefulness of IRS and LLINs in reducing the density of sandfly vectors significantly.

Joshi et al. [27] studied the impact of IRS with DDT in India, Nepal and Bangladesh and found the spray effective in reducing density of *P. argentipes* for 5 months in Nepal and India. Chowdhury et al. [49] while reviewing the performance of IRS in India and Nepal in the context of VL elimination found that after two weeks, four weeks and 5–6 months of DDT spraying in India (Vaishali district) and Nepal (Sunsari district), the percentage mortality of *P. argentipes* in cone bioassay on wall surfaces revealed 70 versus 100 %, 50 versus 55 % and 20 versus 25 % mortality in India and Nepal respectively.

Vector control in Bangladesh, which was previously deficient [50], has improved markedly. New approaches such as the use of slow-release insecticides and KO-Tab123 for impregnation of nets were reported to be highly satisfactory [51].

Picado et al. [52] reviewed the impact of vector control in Southeast Asia. They observed that indoor residual spraying and use of treated nets have low effectiveness, which warrants improvement in the quality of spraying, and research on alternative, integrated vector control methods to achieve VL elimination.

Recently, Coleman et al. [53] reported spraying of DDT (1gm/m^2^) on walls up to 84.9 % and concluded that DDT-based IRS is suboptimal for achieving the goal of VL elimination.

In addition to Southeast Asian countries, Alexander and Maroli [54] while reviewing the susceptibility status of *P. papatasi* in 2003 reported tolerance to DDT, methoxychlor and dieldrin in Egypt, the Islamic Republic of Iran and Israel; *Lutzomyia youngi* were tolerant to malathion and fenthion while resistant to propoxur and deltamethrin. *Lutzomyia longipalpis* was tolerant to fenitrothion and pirimiphos-methyl. High resistance in *P. papatasi* against malathion and propoxur was reported in Sudan [55].

### Collateral benefits of anti-malaria programme in VL control

In Southeast Asia, no separate national programme existed for control of VL; rather, IRS was undertaken for control of malaria vectors. The anti-malaria operations helped VL control as observed by Sanyal et al. [14] in 1979 that apparent disappearance of VL between 1960 and 1970 may partly be as a collateral benefit of DDT spraying under the National Malaria Eradication Programme. In 1994, *P. argentipes* was reported to be absent from Kamrup district of Assam (India), due to continuous spraying of insecticides in high *Plasmodium falciparum* areas [56]. Pandya [57] also observed the impact of malathion spraying in checking the population of *P. argentipes* for 8–9 months in Surat district in the state of Gujarat (India).

In the Islamic Republic of Iran, Nadim and Amini observed that DDT spraying for malaria control significantly reduced the incidence of CL [58]. Phlebotomids were rarely caught from walls but transmission could not be interrupted possibly due to the sleeping habits of people.

## Conclusions and way forward

The review of the literature on the susceptibility of sandflies in Southeast Asia reveals that *P. argentipes*, the major vector of VL, has developed resistance to DDT in areas ofprevious use such as in the states of Bihar, Jharkhand and Maharashtra and parts of West Bengal. In areas where cases of kala-azar have been recently reported such as eastern Uttar Pradesh in India, the vector is reported to be susceptible to DDT andthe same is true for some areas of West Bengal, although further verification is required in these areas. In Gadchiroli district, Maharashtra, India, where synthetic pyrethroids have been used for a long time for malaria control, co-prevalent populations of *P. argentipes* are no longer susceptible to these insecticides. However, because *P. argentipes* is resistant to DDT in important areas of kala-azar endemicity in India where pyrethroid insecticides have not been used previously, these insecticides should be used against kala-azar vectors as part of the insecticide resistance management strategy. There is need to generate more data on insecticide susceptibility of vector species to insecticides in Bangladesh and Nepal. There is also a need to establish vector surveillance in the disease-free areas in previously kala-azar endemic countries or states.

*Phlebotomus papatasi* has developed resistance to DDT, but currently there is little public health problem owing to the very low incidence of CL in the region. However, with changing ecological and climatic conditions, there should be preparedness for alternative tools. In order to manage the resistance in sandflies generally, use of rotation, mosaics and mixtures of insecticides with unrelated modes of action [59] are worth attempting to delay the development of resistance in areas that remain susceptible. There are only a few reports on the mechanism of insecticide resistance in sandflies [44, 60], necessitating further studies on management of resistance.

In this regard, it is noteworthy that the National Vector-borne Disease Control Programme of India has initiated a pilot project in Bihar to evaluate the effectiveness of alpha-cypermethrin indoor residual spraying on kala-azar replacing the use of DDT. To support this effort, a training of trainers was organized in November 2015 in collaboration with the World Health Organization on the correct procedure of indoor residual spraying including the introduction of hand compression sprayers.

There is a need to conduct a comprehensive study on the distribution and type of insecticide resistance mechanisms in sandflies, strengthen public health entomology capacity including a system for collection of resistance data from the field, monitoring and GIS-based mapping of resistance, financial provision of susceptibility test kits and supplies, and training of programme managers in insecticide resistance management. In order to manage insecticide resistance in sandflies and other vectors of VL and CL, use of rotation, mosaics and mixtures of insecticides are possible approaches worth exploring [44, 59, 60].

Finally, capacity strengthening is required not only in India where kala-azar has yet to be eliminated but also in the neighbouring endemic countries of Bangladesh, Bhutan, Nepal andSri Lanka as part of vector surveillance within an integrated vector management approach.

## Additional file

Additional file 1: Multilingual abstracts in the five official working languages of the United Nations. (PDF 461 kb)

## Abbreviations

CL: Cutaneous leishmaniasis
DDT: Dichlorodiphenyltrichloroethane
IRS: Indoor residual spraying
ITN: Insecticide treated net
LLIN: Long-lasting insecticidal net (as a product)
LN: Long-lasting insecticidal net (as a formulation)
VL: Visceral leishmaniasis

## References

[1] Swaminath CS, Short HE, Anderson LAP (1942). Transmission of Indian kala-azar to man by the bite of *P. argentipes* Ann and Brun. Ind J Med Res.

[2] Alvar J, Vélez ID, Bern C, Herrero M, Desjeux P (2012). Leishmaniasis worldwide and global estimates of its incidence. PLoS One.

[3] Joshi GC, Kaul SM, Wattal BL (1979). Susceptibility of sandflies to organochlorine insecticides in Bihar (India). J Commun Dis.

[4] Mukhopadhyay AK, Saxena NBL, Narsimham MVVL (1990). Susceptibility of *Phlebotomus argentipes* to DDT in some kala-azar endemic areas of Bihar (India). Indian J Med Res.

[5] Naik SR, Rao PN, Datta DV, Mehta SK, Mahajan RC (1979). Kala-azar in north-western India: a study of 23 patients. Trans R Soc Trop Med Hyg.

[6] Sharma NL, Mahajan VK, Kanga A, Sood A, Katoch VM (2005). Localized cutaneous leishmanisis due to *Leishmania donovani* and *Leishmania tropica*: Preliminary. Trans R Soc Trop Med Hyg.

[7] Yangzom T, Cruz I, Bern C, Argaw D, den Boer M (2012). Endemic transmission of visceral leishmaniasis in Bhutan. Am J Trop Med Hyg.

[8] Uranw S, Hasker E, Roy L, Meheus F, Das ML (2013). An outbreak investigation of visceral leishmaniasis among residents of Dharan town, eastern Nepal, evidence for urban transmission of *Leishmania donovani*. BMC Infect Dis.

[9] Kariyawasam KK, Edirisuriya CS, Senerath U, Hensmen D, Siriwardana HV (2015). Characterisation of cutaneous leishmaniasis in Matara district, southern Sri Lanka: evidence for case clustering. Pathog Glob Health.

[10] Sukra K, Kanjanopas K, Amsakul S, Rittaton V, Mungthin M (2013). A survey of sandflies in the affected areas of leishmaniasis, southern Thailand. Parasitol Res.

[11] Ranganathan S, Swaminathan S (2015). Sandfly species diversity in association with human activities in the Kani tribe settlements of the Western Ghats, Thiruvananthapuram, Kerala, India. Mem Inst Oswaldo Cruz.

[12] González C, Wang O, Strutz SE, González-Salazar C, Sánchez-Cordero V (2010). Climate change and risk of leishmaniasis in North America: predictions from ecological niche models of vector and reservoir species. PLoS Negl Trop Dis.

[13] World Health Organization (2005). Regional Technical Advisory Group on Kala-azar Elimination. Report of the first meeting, Manesar.

[14] Sanyal R, Banerjee DP, Ghosh TK, Ghose JN, Misra BS (1979). Longitudinal review of kala-azar in Bihar. J Com Dis.

[15] Sen P (1959). Studies on insecticide resistance insects of public health importance in west Bengal India. Indian J Malariol.

[16] Kaul SM, Wattal BL, Bhatnagar VN, Mathur KK (1978). Preliminary observations on the susceptibility status of *Phlebotomus argentipes* and *P. papatasi* to DDT in two districts of North Bihar (India). J Commun Dis.

[17] World Health Organization (1981). Instructions for determining the susceptibility or resistance of adult blackflies, sandflies and biting, midges to insecticides. (WHO/VBC document 81.810).

[18] Dhanda V, Shetty PS, Dhiman RC, Mahajan RC (1983). Studies on phlebotomine sandflies as vectors of kala-azar in Bihar. Proc. Indo-UK Workshop on Leishmaniasis.

[19] Das Gupta RK, Saxena NBL, Joshi RD, Rao JS (1995). DDT resistance in P. papatasi in Bihar. J Commun Dis.

[20] Dhiman RC, Mittal PK (2000). A note on susceptibility status of *Phlebotomus papatasi* (Scopoli) population to insecticides. J Commun Dis.

[21] Thapar BR, Joshi RD, Rao JS, Saxena NBL (1993). Susceptibility status of *Phlebotomus papatasi* (Scopoli) (Diptera: Psychodidae) to chlorinated hydrocarbons in Panchmahal district of Gujarat state (India). J Commun Dis.

[22] Bansal SK, Singh KV (1996). Susceptibility status of *Phlebotomus papatasi* and *Sergentomyia punjabensis* (Diptera: Psychodidae) to some insecticides in district Bikaner (Rajasthan). J Commun Dis.

[23] Singh KV, Bansal SK (1996). Insecticide susceptibility of *Phlebotomus papatasi* to organochlorine, organophosphate, and carbamate compounds in some arid areas of Western Rajasthan. Indian J Med Res.

[24] Mukhopadhyay AK, Chakravarty AK, Kureel VR, Shivaraj (1987). Resurgence of *Phlebotomus argentipes* and *Ph. papatasi* in parts of Bihar (India) after DDT spraying. Indian J Med Res.

[25] Kaul SM, Das RK, Shivraj, Saxena NBL, Narsimham MVVL (1993). Entomological monitoring of kala-azar control in Bihar state India: observations in Vaishali and Patna district. J Commun Dis.

[26] Kaul SM, Sharma RS, Dey KP, Rai RN, Verghese T (1994). Preliminary observations on impact of DDT spraying on a population of *Phlebotomus argentipes* in Varanasi district Uttar Pradesh. Bull World Health Organ.

[27] Joshi AB, Das ML, Akhter S, Chowdhury R, Mondal D (2009). Chemical and environmental vector control as a contribution to the elimination of visceral leishmaniasis on the Indian subcontinent: cluster randomized controlled trials in Bangladesh, India and Nepal. BMC Med.

[28] Joshi RD, Rai RN (1994). Impact of DDT spraying on populations of *P. argentipes* and *P. papatasi* in Varanasi district, Uttar Pradesh. J Commun Dis.

[29] Mukhopadhyay AK, Hati AK, Chakraborty S, Saxena NBL (1996). Effect of DDT on Phlebotomus sandfly in kala-azar endemic foci in West Bengal. J Commun Dis.

[30] NMEP Annual Report of the National Malaria Eradication Programme. Ministry Health and Family Welfare of the Government of India; 1991.

[31] Basak B, Tandon N (1995). Observations on susceptibility status of *Phlebotomus argentipes* to DDT in District South 24-Paraganas, West Bengal. J Commun Dis.

[32] Chandra G, Bhattacharya J, Hati AK (1995). Susceptibility status of *Phlebotomus argentipes* to DDT, dieldrin and malathion in Hoogly, West Bengal. J Commun Dis.

[33] Kumar V, Shankar L, Kesari S, Bhunia GS, Dinesh DS (2015). Insecticide susceptibility of *Phlebotomus argentipes* & assessment of vector control in two districts of West Bengal, India. Indian J Med Res.

[34] Amalraj DD, Sivagnaname N, Srinivasan R (1999). Susceptibility of *Phlebotomus argentipes* and *P. papatasi* (Diptera: Psychodidae) to insecticides. J Commun Dis.

[35] Singh R, Das RK, Sharma SK (2001). Resistance of sandflies to DDT in Kala-azar endemic districts of Bihar in India. Bull World Health Organ.

[36] Dhiman RC, Raghavendra K, Kumar V, Kesari S, Kishore K (2003). Susceptibility status of *Phlebotomus argentipes* to insecticide in districts Vaishali and Patna. J Commun Dis.

[37] Dinesh DS, Das ML, Picado A, Roy L, Rijal S (2010). Insecticide susceptibility of *Phlebotomus argentipes* in visceral leishmaniasis endemic districts in India and Nepal. PLoS Negl Trop Dis.

[38] Singh R, Kumar P (2015). Susceptibility of the sandfly *Phlebotomus argentipes* Annandale and Brunetti (Diptera: Psychodidae) to insecticides in endemic areas of visceral leishmaniasis in Bihar, India. Jpn J Infect Dis.

[39] Singh RK, Mittal PK, Dhiman RC (2012). Susceptibility status of *Phlebotomus argentipes* vector of visceral leishmaniasis to insecticides in different foci in three states of India. Jour Vec Borne Dis.

[40] Rao JS, Sharma SK, Bhattacharya D, Saxena NBL (2001). Sand fly survey in Nainital and Almora, district of Uttranchal with particular reference to *Phlebotomus argentipes* vector of kala-azar. J Commun Dis.

[41] Choudhury S. Kala-azar in Bangladesh [Paper presented at Inter-Country Workshop on cross-border issues in malaria, kala-azar and Japanese encephalitis prevention and control]. Hetauda, Nepal; July 25–28 2000.

[42] Environmental Health Project Susceptibility status of sand fly in Dhanusa District. Nepal (unpublished document); 2001.

[43] Anonymous. The annual internal assessment of malaria and kala-azar control activities 2000. Epidemiology and Disease Control Division, Department of Health Services, Ministry of Hetauda.2000; His Majesty’s Government of Nepal and Vector Borne Disease Research and Training Centre, November 2000, Kathmandu, Nepal.

[44] Surendran SN, Karunaratne SH, Adams Z, Hemingway J, Hawkes NJ (2005). Molecular and biochemical characterization of a sand fly population from Sri Lanka: evidence for insecticide resistance due to altered esterases and insensitive acetyl cholinesterase. Bull Entomol Res.

[45] Dinesh DS, Das P, Picado A, Davies C, Speybroeck N (2008). Long-lasting insecticidal nets fail at household level to reduce abundance of sandfly vector *Phlebotomus argentipes* in treated houses in Bihar (India). Trop Med Int Health.

[46] Picado A, Das ML, Kumar V, Kesari S, Dinesh DS (2010). Effect of village-wide use of long-lasting insecticidal nets on visceral leishmaniasis vectors in India and Nepal: a cluster randomized trial. PLoS Negl Trop Dis.

[47] Das ML, Roy L, Rijal S, Paudel IS, Picado A (2010). Comparative study of kala-azar vector control measures in eastern Nepal. Acta Trop.

[48] Kumar V, Kesari S, Kumar AJ, Dinesh DS, Ranjan A (2009). Vector density and the control of kala-azar in Bihar, India. Mem Inst Oswaldo Cruz.

[49] Chowdhury R, Huda MM, Kumar V, Das P, Joshi AB (2011). The Indian and Nepalese programmes of indoor residual spraying for the elimination of visceral leishmaniasis: performance and effectiveness. Ann Trop Med Parasitol.

[50] Mondal D, Alam MS, Karim Z, Haque R, Boelaert M (2008). Present situation of vector-control management in Bangladesh: a wake up call. Health Policy.

[51] Mondal D, Chowdhury R, Huda MM, Maheswary NP, Akther S (2010). Insecticide-treated bed nets in rural Bangladesh: their potential role in the visceral leishmaniasis elimination programme. Trop Med Int Health.

[52] Picado A, Das AP, Bhattacharya S, Boelaert M (2012). Vector control interventions for visceral leishmaniasis elimination initiative in South Asia, 2005–2010. Indian J Med Res.

[53] Coleman M, Foster GM, Deb R, Pratap Singh R, Ismail HM (2015). DDT-based indoor residual spraying suboptimal for visceral leishmaniasis elimination in India. Proc Natl Acad Sci U S A.

[54] Alexander B, Maroli M (2003). Control of phlebotomine sandflies. Med Vet Entomol.

[55] Hassan MM, Widaa SO, Osman OM, Numiary MS, Ibrahim MA (2012). Insecticide resistance in the sand fly, *Phlebotomus papatasi* from Khartoum State, Sudan. Parasit Vectors.

[56] Kaul SM, Sharma RS, Borgohain BK, Das NS, Verghese T (1994). Absence of *Phlebotomus argentipes* Ann & Brun. (Diptera: Psychodidae) the vector of Indian kala-azar from Kamrup district, Assam. J Commun Dis.

[57] Pandya AP (1983). Impact of antimalaria house spraying on phlebotomid population in Surat district, Gujarat. Indian J Med Res.

[58] Nadim A, Amini H (1970). The effect of antimalarial spraying on the transmission of zoonotic cutaneous leishmaniasis. Trop Med Geogr.

[59] Hemingway J, Ranson H (2000). Insecticide resistance in insect vectors of human disease. Annu Rev Entomol.

[60] El-Sayed S, Hemingway J, Lane RP (1989). Susceptibility baselines for DDT metabolism and related enzyme systems in the sand fly *Phlebotomus papatasi* (Scopoli) (Diptera: Psychodidae). Bull Entomol Res.

