# Supplementary material for: Insecticide resistance in phlebotomine sandflies in Southeast Asia with emphasis on the Indian subcontinent
Source: Infect Dis Poverty. 2016 Nov 7;5:106. doi: 10.1186/s40249-016-0200-3 (PMC5098277; doi:10.1186/s40249-016-0200-3)
Supplement: Additional file 1: — Multilingual abstracts in the five official working languages of the United Nations. (PDF 461 kb) [file 40249_2016_200_MOESM1_ESM.pdf]

Translation of the abstract into the five official working languages of the United Nations

مقاومة المبيدات الحشرية لدى ذباب الرمل الفاصد في جنوب شرق آسيا مع التركيز على شبه القارة الهندية

راميش سي ديمان وراجبال إس ياداف

تلخيص

معلومات عامة: يُعد داء الليشمانيات الحشوي، المعروف باسم الكلازار في الهند، هو مشكلة صحية عامة عالمية. وينتشر هذا الداء الوبائي في جنوب شرق آسيا، وبنغلاديش، وبوتان، والهند، ونيبال، وسريلانكا وتايلاند. وجرى تأكيد دور ذباب الرمال بوصفه ناقل الكلازار لأول مرة في عام 1942 في الهند. وقد أبلغ أناندالي وبرونيتي لأول مرة عن مقاومة المبيدات الحشرية لدى *الفواصد*، ناقلة داء الكلازار في شبه القارة الهندية، في عام 1987 في ولاية بيهار، الهند. وتنص هذه المادة على مراجعة تحديد نطاق الدراسات التي أجريت 1959-2015 على مقاومة المبيدات الحشرية لدى *الفواصد والفواصد البابتيسية* (سكوبولي), ناقلات داء الليشمانيات الحشوي والجلدي على التوالي، في جنوب شرق آسيا، لا سيما في بنغلاديش والهند ونيبال و سيريلانكا.

النتائج: وأفادت الدراسات التي أجريت في مناطق بولاية بيهار والبنغال الغربية في الهند حيث يتوطن داء الكلازار عن مقاومة *الفواصد* للمبيدات الحشرية, بينما تم الإبلاغ في المناطق غير الموبوءة عن أنها قد تكون عرضة لذلك. وفي مناطق بنيبال المجاورة للهند، توجد مؤشرات على مقاومة للمبيدات الحشرية؛ حيث جرى الإبلاغ عن المقاومة الكيميائية الحيوية في سري لانكا. وبالرغم من عدم إجراء دراسات معملية في بنغلاديش، فقد جرى الإبلاغ عن أن ناقل ذبابة الرمل مازال مشكوك في أنها عرضة للابيرثرويدات في جميع المناطق الموبوءة بهذا الداء في الدول سالفة الذكر.

الاستنتاجات: ثمة حاجة لإجراء دراسات لتحديد مقاومة ناقلات ذبابة الرمل لجميع الطبقات المتاحة من المبيدات الحشرية المحتملة في المناطق الموبوءة بذلك الداء. كذلك ثمة حاجة لتقييم تأثير الرش الثمالي داخل المباني بالمبيدات الحشرية والبيرثرويدات في حالة الكلازار في الهند حيث مازالت 54 منطقة من مناطق توطن المرض تعتبر مناطق موبوءة، وتعزيز قدرات المراقبة الحشرية، ووضع خطة إدارة مبيدات الآفات وتنفيذها. وقد تم إدخال الرش الثمالي بألفا سايبرمثرين داخل المباني في 33 منطقة من المناطق الموبوءة بالكلازار في ولاية بيهار في الهند في تجربة استرشادية؛ وينبغي استخدام النتائج لاتخاذ قرارات عليمة بشأن توسيع نطاق التغطية باستخدام ألفا سايبرمثرين في جميع المناطق الموبوءة الأخرى لتحقيق الهدف المُعدل بالقضاء على داء الليشمانيات الحشوي بحلول عام 2020.

Translated from English version into Arabic by Fathia Sobhi, through

TRANSLATORS
WITHOUT BORDERS

南亚地区特别是印度次大陆白蛉杀虫剂抗性

Ramesh C. Dhiman and Rajpal S. Yadav

摘要

引言：内脏利什曼病在印度俗称黑热病，是一个全球性的公共卫生问题。内脏利什曼病在南亚、孟加拉国、不丹、印度、尼泊尔、斯里兰卡和泰国流行。白蛉作为黑热病的传播媒介于1942 年首次在印度得到证实。1987 年关于印度次大陆黑热病的传播媒介——银足白蛉（*Phlebotomus argentipes* Annandale and Brunetti）的抗药性首次报道于印度比哈尔。本文对1959-2015 年银足白蛉和静食白蛉[*P. papatasi*（Scopoli）]杀虫剂抗药性研究进行了综述，这两种白蛉分别是南亚地区内脏和皮肤利什曼病的传播媒介，主要分布在孟加拉国、印度、尼泊尔和斯里兰卡。

结果：在印度比哈尔和西孟加拉邦黑热病流行区报道了静食白蛉对 DDT 存在抗性，而在非流行区，白蛉对杀虫剂是敏感的。在尼泊尔与印度接壤的地区，有 DDT 抗性存在的迹象，而且在斯里兰卡已有生化抗性。在孟加拉国仍未开展过实验室研究。然而，据报道在上述黑热病流行国家，白蛉对拟除虫菊酯类杀虫剂仍敏感。

结论：研究需要确定在黑热病流行区媒介白蛉对所有可用的、潜在的杀虫剂的抗性状况。需要评估印度 54 个黑热病流行区室内滞留喷洒 DDT 和拟除虫菊酯的影响，加强媒介监测能力，并制定和实施农药管理计划。在一项试点研究中，印度比哈尔州的 33 个黑热病流行区已采用高效氯氰菊酯室内滞留喷洒，该项措施的结果将用于帮助决定可否将高效氯氰菊酯喷洒的范围扩展至其他所有流行区，以实现 2020 年对内脏利什曼病的消除。

Translated from English version into Chinese by Feng Xin-Yu, edited by Pin Yang

CDC CHINESE CENTER FOR DISEASE CONTROL AND PREVENTION
NATIONAL INSTITUTE OF PARASITIC DISEASES

Résistance aux insecticides chez les phlébotomes dans l’Asie du Sud-Est avec concentration sur le sous-continent indien

Ramesh C. Dhiman et Rajpal S. Yadav

Résumé

Contexte : la leishmaniose viscérale, couramment appelée kala-azar en Inde, constitue un problème de santé publique mondial. En Asie du Sud-Est, le Bangladesh, le Bhoutan, l’Inde, le Népal, le Sri Lanka et la Thaïlande sont des régions où la leishmaniose viscérale est endémique. Le rôle des phlébotomes en tant que vecteur du kala-azar a été confirmé pour la première fois en 1942 en Inde. La résistance aux insecticides chez le *Phlebotomus argentipes* Annandale et Brunetti, le vecteur du kala-azar dans le sous-continent indien, a été signalée pour la première fois en 1987 dans l’État de Bihar en Inde. Le présent article fournit une revue exploratoire des études entreprises de 1959 à 2015 sur la résistance aux insecticides des *P. argentipes* et *P. papatasi* (Scopoli), étant respectivement les vecteurs de la leishmaniose viscérale et cutanée, dans l’Asie du Sud-Est, principalement au Bangladesh, en Inde, au Népal et au Sri Lanka.

Résultats : les études réalisées dans les régions de Bihar et du Bengale occidental en Inde, là où le kala-azar est endémique, ont fait état de la résistance du *P. argentipes* au DDT, tandis que sa sensibilité à cette même substance a été signalée dans les zones non endémiques. Dans les régions du Népal limitrophes de l'Inde, certains signes indiquent une résistance au DDT et des cas de résistance biochimique ont été rapportés au Sri Lanka. Aucune étude de laboratoire n'a été entreprise au Bangladesh. Néanmoins, le phlébotome en tant que vecteur est considéré comme étant toujours sensible aux pyréthroïdes dans toutes les zones où le kala-azar est endémique dans les pays mentionnés ci-dessus.
Conclusions : des études doivent être réalisées afin de déterminer la résistance des phlébotomes en tant que vecteur à toutes les classes disponibles d'insecticides potentiels dans les zones où le kala-azar est endémique. Il est non seulement nécessaire d'évaluer l'impact de la pulvérisation de DDT et de pyréthroïdes à effet rémanent à l'intérieur des habitations sur l'incidence du kala-azar en Inde où la maladie reste endémique dans 54 districts, mais aussi de renforcer les capacités de surveillance entomologique et de développer et mettre en œuvre un plan de gestion des insecticides. La pulvérisation d'alpha cyperméthrine à effet rémanent à l'intérieur des habitations a été introduite dans le cadre d'un projet pilote dans 33 districts de l'État de Bihar en Inde où le kala-azar est endémique. Les résultats devraient être utilisés afin d'éclairer les décisions relatives à l'extension de la couverture de l'alpha cyperméthrine à tous les autres districts restants où la maladie est endémique afin de satisfaire l'objectif révisé d'élimination de la leishmaniose viscérale d'ici 2020.

Translated from English version into French by eric ragu, through

TRANSLATORS
WITHOUT BORDERS

Резистентность к действию инсектицидов на москитов рода Флеботомус в Юго-Восточной Азии с акцентом на Индийский субконтинент

Рамеш К. Дхиман и Раджпал С. Ядав

Резюме

Краткая информация: Висцеральный лейшманиоз, также известный, как кала-азар в Индии – общемировая проблема, угрожающая здоровью населения. Заболевание является высокоэндемичным для стран Юго-Восточной Азии, Бангладеша, Бутана, Индии, Непала, Шри Ланки и Таиланда. Роль москитов в качестве переносчиков и возбудителей кала-азар была подтверждена в 1942 году в Индии. Инсектицидная резистентность *Phlebotomusargentipes*Annandale и Brunetti - у возбудителей кала-азар на Индийском субконтиненте впервые была выявлена в 1987 в Штате Бихар, Индия. Данная статья посвящена анализу исследований, выполненных с 1959 по 2015 годы, инсектицидной резистентности *P. argentipes* и *P. papatasi* (Scopoli), возбудителей висцерального и кожного лейшманиоза соответственно в Юго-Восточной Азии, а именно в Бангладещ, Индии,

Непале и Шри-Ланке.
Результаты: Исследования, проведенные в Штате Бихар и Западной Бенгалии (Индия), эндемичных по кала-азар, подтвердили резистентность *P. Argentipes* к DDT, в то время как в неэндемичных районах резистентность носит предположительный характер. На территориях Непала, граничащих с Индией, есть признаки резистентности к DDT; о биохимической резистентности сообщается в Шри Ланке. В Бангладеш лабораторные исследования не проводились; однако, переносчики-москиты, предположительно, все еше чувствительны к пиретроиду, применяемому в инсектицидных средствах для предотвращения кала-азар на всех эндемичных территориях вышеназванных стран.
Выводы: Для определения резистентности возбудителей к инсектицидам на территориях, эндемичных по кала-азар, требуются исследования. Необходимо оценить эффект от применения внутри помещений спреев с DDT и пиретроидами, влияние на количество случаев заражения кала-азар в Индии, где 54 района остаются эндемичными по данному заболеванию, усилить меры энтомологического контроля, разработать и применить план по использованию инсектицидов. В качестве пилотного проекта в 33 эндемичных по кала-азар районах Штата Битар в Индии стали применяться спреи с альфа-циперметрином внутри помещений; результаты предстоит оценить для принятия решений о широкомасштабном применении альфа-циперметрина в оставшихся эндемичных районах для достижения цели по предотвращению висцерального лейшманиоза к 2020 году.

Translated from English version into Russian by Ms Zhdanova, through

TRANSLATORS
WITHOUT BORDERS

Resistencia a los insecticidas en mosca de arena Phlebotominae en el sudeste asiático, con énfasis en el subcontinente indio

Ramesh C. Dhiman and Rajpal S. Yadav

Resumen

Antecedentes: La leishmaniasis visceral, comúnmente conocida como kala-azar en la India, es un problema de salud pública mundial. En el sudeste de Asia, Bangladesh, Bhután, India, Nepal, Sri Lanka y Tailandia la leishmaniasis visceral es endémica. El papel de las moscas de arena como el vector de kala-azar se confirmó por primera vez en 1942 en la India. La resistencia a los insecticidas en *Phlebotomus argentipes* Annandale y Brunetti, el vector de kala-azar en el subcontinente indio, fue reportado por primera vez en 1987 en Bihar, India. Este artículo proporciona una revisión del alcance de los estudios llevados a cabo desde 1959 hasta 2015 sobre la resistencia a los insecticidas en *P. argentipes* y *P. papatasi* (Scopoli), los vectores de la leishmaniasis visceral y cutánea, respectivamente, en el sudeste de Asia, sobre todo en Bangladesh, India, Nepal y Sri Lanka.
Resultados: Los estudios realizados en zonas de Bihar y Bengala Occidental en la India, donde la

kala-azar es endémica han reportado resistencia de *P. argentipes* al DDT, mientras que en las zonas no endémicas se ha informado de que es susceptible. En las zonas de Nepal bordeando la India, hay indicios de resistencia al DDT; resistencia bioquímica ha sido reportada en Sri Lanka. No se han llevado a cabo estudios de laboratorio en Bangladesh; sin embargo, el vector de la mosca de la arena se ha reportado que es susceptible a los pyrethroids en todas las zonas endémicas de kala-azar en los países antes mencionados.

Conclusiones: Se necesitan estudios para determinar la resistencia de los vectores de la mosca de arena a todos los tipos de insecticidas potenciales disponibles en las áreas endémicas de kala-azar. Hay necesidad de evaluar el impacto de la fumigación de interiores con DDT y pyrethroids en la incidencia de kala-azar en la India, donde 54 distritos siguen siendo endémicos para la enfermedad, de fortalecer la capacidad de vigilancia entomológica y desarrollar e implementar un plan de gestión de insecticidas. La fumigación de interiores Alfa-cipermetrina se ha introducido en 33 distritos endémicos de kala-azar en el estado de Bihar de la India en una prueba piloto.

Los resultados deberían utilizarse para fundamentar las decisiones en ampliar la cobertura con alpha-cypermethrin en todos los distritos endémicos restantes para lograr la meta revisada de la eliminación de la leishmaniasis visceral en 2020.

Translated from English version into Spanish by patriciacassoni, through

TRANSLATORS
WITHOUT BORDERS
